# Medical Opinion and Movement

**Published:** 1911-03-25

**Authors:** 


### Medical Opinion and Movement.

### Varicella Micro-organism.

At a recent meeting of the Société de Biologie Drs. Magnan and de la Riboisière gave an account of their bacteriological researches in cases of varicella. They describe a bacillus measuring 1.1 to 2.2$\mu$ long by 0.2 to 0.3 $\mu$ wide. They have always been able to detect it in the vesicles of varicella. The bacilli are often arranged in palisades and form small groups which are quite characteristic. They are generally in pairs, sometimes side by side, sometimes end to end, frequently joined at an angle more or less acute so as to form a v or an accent circumflex. Their extremities are rounded. These bacilli are very numerous one to two hours after the appearance of the eruption, when there are only two or three vesicles. On the third day of the eruption their number diminishes considerably, and from the fifth day it is almost impossible to detect them. The authors have altogether failed in their attempts to cultivate this bacillus, although they have tried all kinds of media likely to be favourable for such a micro-organism.

### Regeneration of Osseous-tissue.

The repair of bony tissues and the manner in which re-ossification takes place has been the subject of a good deal of study recently with a view to discover the causes of failure of re-ossification in certain cases of fracture, and to find means by which the natural reparative processes might be assisted. The fact that ossification may take place in almost any tissue, including notably muscular and connective tissue, suggests that the regeneration of bone may take place under suitable conditions apart from its usual formation from the periosteum and bone marrow. It has been shown that heteroplastic ossification may take place even in the kidneys. Sacerdotti and Frattin found that after ligature of the renal vessels in guinea-pigs, the kidneys developed, in those surviving more than three months, complete osseous-tissue with marrow and osteoblasts. Whatever these facts may signify, for the surgeon, at any rate for the present, the preservation of the periosteum and its careful adjustment in all cases of operative interference are of primary importance in securing a satisfactory regeneration of the bony structures. From a series of observations in cases of osteomalacia, in which it has been claimed by Carnot and Slavu that the administration of adrenalin led to improvement in the condition, it has been suggested that this drug might also assist in the reparation of healthy bone. Dr. Vignier reports two cases of fracture in which adrenalin was administered with satisfactory results, as owing to the nature of the injuries successful reunion appeared doubtful. Dr. Pochhammer suggests that the reparative processes may be assisted in cases of surgical intervention by spreading blood clots around the bony lesion. He is of opinion that they excite reaction by causing irritation of the adjacent periosteum.

### Sugar in the Urine.

Dr. Gosta Bohmansson has recently carried out some researches to determine the most reliable qualitative test for sugar in the urine. He gives the preference to Almen's reaction with bismuth oxide. Fehling's test is unreliable because reduction may take place in the presence of other substances than sugar in the urine, or in the presence of others again the precipitate may redissolve. On the other hand, the bismuth reaction is not affected by uric acid or creatinin and the reduced bismuth is not liable to be redissolved. Positive reactions may, however, occur with Almen's test in the absence of sugar, and, according to Dr. Bohmansson, this is due to the colouring matters in the urine, notably the urochrome. In order to get rid of these the author first treats the urine with hydrochloric acid and animal charcoal. His method of procedure is as follows: To 10 cubic centimetres of urine are added one-fifth of the volume of 25 per cent. hydrochloric acid and an equal volume of moist animal charcoal. The mixture is well shaken for a minute and then filtered. The filtrate is neutralised with a few drops of strong soda solution and then the test of Almen applied. Specimens of urine from twenty patients suffering from liver or heart disease all gave a positive reaction with Almen's test in the ordinary way. After treating with hydrochloric acid and charcoal only five gave a positive reaction, and these five were found to contain a pathological quantity of sugar.

### Colocynth as a Purgative.

Dr. J. H. Padtberg has examined the effect of colocynth upon gastric and intestinal movements by means of the *x*-rays in a manner similar to that employed by other observers to determine the mode of action of purgatives on the digestive tract. His observations were carried out on cats. After fasting for twenty-four hours they were given a meal of potato containing 5 grammes of bismuth hydrate. Ten cubic centimetres of a 10 per cent. decoction of colocynth was then given by a stomach tube a quarter of an hour after the meal. An abundant loose stool resulted one to four hours later, which was repeated two or three times. Under ordinary conditions the stomach of the cat is emptied in three hours. After the colocynth the stomach is emptied in an hour to an hour and a half. The first part of the intestinal contents reaches the colon in half an hour after the meal, whereas the usual time is about three hours. The stomach contents may even traverse the whole of the small intestine in a quarter of an hour. The whole of the small intestine was empty after four hours, whereas under normal conditions the products of digestion may still be there seven hours after a meal. When the bismuth meal reaches the colon the usual antiperistaltic movements of this segment cease. This peculiarity has been noted with other vegetable laxatives, but is not observed with the salines. On the other hand, the arrival of the colocynth in the large intestine does not at once excite defæcation, as in the case of senna, but the activity of the lower bowel is much increased and the evacuation takes place in the course of about an hour and a half. Under normal conditions the progress of the contents along the large intestine is so slow that it can hardly be appreciated on the radioscopic screen. It takes twelve hours or more.

### The Treatment of Typhoid Carriers.

The difficult problem of curing the typhoid carrier has on many occasions within the last two or three years baffled the physicians in charge of one of these dangerous disseminators of infection. In the *Medical Review* is a suggestion based upon the researches of H. Conradi. This author gave intravenous injections of typhoid bacilli to rabbits, in sub-lethal doses. In ten days all the rabbits had typhoid bacilli in the liver, gall-bladder, heart, and bone-marrow; the kidneys, spleen, and urine also contained bacilli in many cases. At the end of two weeks the gall-bladder alone was found always to contain them, and then 5 c.c. of a mixture of 5 parts milk, 4 parts cream, and 1 part chloroform were administered *per rectum* daily. Transient narcosis followed each injection; but in this ten days sixteen out of twenty-one rabbits no longer contained bacilli, while twelve control-animals continued to exhibit them. Further experiments served to support the view that chloroform can sterilise the gall-bladder, and the possibility of using this drug to cure human typhoid carriers seems one distinctly worth testing.

### Oculomotor Polioencephalitis.

In view of the extensive and serious epidemics of poliomyelitis and polioencephalitis which have attracted attention in America and elsewhere during the last year or two, a special interest attaches to certain conclusions expressed by Mr. Sydney Stephenson in the *Ophthalmoscope*. He maintains a series of propositions which may be briefly summarised thus: There is a particular form of paralytic strabismus in children which is due to polioencephalitis. It is not uncommon, and is most frequent in children under one year of age. It is associated comparatively seldom with other symptoms indicative of cerebral disorder. Zymotic diseases appear to be important factors in its causation. Although the paralysis may affect any of the extrinsic muscles of the eyeball, yet in three-fourths of the cases the external rectus muscle is alone involved. The intrinsic musculature is seldom attacked. The common form of encephalic strabismus is very apt to be confused with the ordinary form of concomitant convergent strabismus. The author says that he is not prepared to suggest any means whereby the two conditions can be distinguished in the absence of a trustworthy history of the case, except by electrical examination of the affected muscles, no simple matter when dealing with the eyes of a young child.

### Rice Diet in Skin Diseases.

The value of a restricted and purely vegetarian diet in certain types of acute skin disease is the subject of an article in the *Medical Record* by Dr. Bulkley. The chief ingredient of the diet is rice boiled in water; this is recommended to be flaked somewhat by leaving it uncovered on the fire for a while after it has been thoroughly cooked. Nothing else is allowed but bread, butter, and water; the rice is to be eaten slowly on a fork with abundance of butter and salt, and is to be thoroughly masticated. This diet may be adopted with the greatest success, says the author, for acute eczema, psoriasis, bullous forms of erythema, and so on. It should not be ordered for more than a week; at the end of that time a gradual return to ordinary light diet is advisable. No milk is consumed during the actual rice-diet period, but it is allowed freely as soon as the first week of rigorous treatment is over. Dr. Bulkley considers this diet—with appropriate local and medicinal remedies as well—to be indicated for all acute inflammatory and congestive skin lesions, especially those likely to be associated with faulty nitrogenous metabolism. Alcoholics and high livers derive great benefit accordingly. There is no objection to repeating the course of rice diet at intervals.

### Recovery from Optic Atrophy.

In the *Glasgow Medical Journal* Dr. Leslie Buchanan describes recovery under treatment in a number of patients presenting all the signs and symptoms of optic atrophy. The patients have been chiefly males between thirty and fifty years of age, and steady healthy men in responsible positions. Toxic amblyopit, syphilis, alcoholism, renal disease, and such disposing factors were excluded. In each case the complaint was of slowly increasing defect of vision, both for near and distant work. Peripheral contraction of the visual fields, diminution of central vision, and the ophthalmoscopic appearances of early atrophy have been the foundations of the diagnosis. The cases have been watched long enough to exclude nervous diseases; hysteria and malingering have also been negatived. In fact the investigation of the cases from the point of view of ætiology was always fruitless. Under medical treatment—chiefly strychnine and iodide of potassium in liberal doses—a slow but steady improvement began after a month or so; perfect vision and visual fields have been restored, usually in about a year. The appearances of atrophy in the fundus have, however, not been affected The author asks: Are these cases of ordinary atrophy which have been taken in time and prevented from reaching the incurable stage? or are they a special class of case which would be amenable to treatment in any stage? He himself inclines to the former of these two suppositions.

### To Remove a Plaster-of-Paris Bandage.

At a recent meeting of the Austrian Gesellschaft für Physikalische Medizn in Vienna, Dr. Strausky brought forward a very simple and practical expedient for removing plaster-of-Paris bandages without resorting to the use of powerful and expensive special shears. The method is simplicity itself when once the idea has been suggested, and it seems strange that it has not been thought of before: perhaps it has. The method is based on the fact familiar to students of elementary chemistry that calcium carbonate is decomposed by acids with liberation of carbonic acid gas. Dr. Strausky applies this reaction by using vinegar—dilute acetic acid—to soften and destroy the plaster. The line along which it is desired to sever the splint is kept quite wet with vinegar for three minutes or so, and by that time the turns of bandage will be found so softened that an ordinary penknife will easily cut them. After an experience of this simple device which extends over many years, the author of the paper recommends it thoroughly; and if the method will do what he claims for it, beyond doubt it will prove of great service as a time and labour saving expedient.

### The Treatment of Status Epilepticus.

In a curt but admirably thorough paper contributed to the *Medical Chronicle* Dr. A. McDougall defines the aims and methods of treatment for this not very common condition. When confronted with a case that may be *status epilepticus*, the physician's first care must be to exclude puerperal eclampsia and uræmia. Asuming that done, he will be guided by anything he can learn of similar previous attacks. Failing this he must decide from the condition of the patient whether active treatment is or is not wanted. It is always well to begin with a simple enema, which is often enough to end the fits. After that, he can do little harm and may do much good by injecting 30 grains of chloral into the rectum. If after that the fits continue, but without hyperpyrexia, he should have the courage to wait; or if he must do something, let him inject normal saline solution. Even if the patient can swallow, he should have nothing by the mouth for several days, except water. Temperature is a better guide to prognosis than the number of fits; tables are quoted showing that patients with one to four hundred fits a day often recover, while many with fifty to a hundred die. Hyperpyrexia, though not necessarily fatal, is a grave warning and calls for prompt treatment. It is also important to observe the depth of coma between successive fits: if the corneal reflex returns or the patient, though unconscious, can swallow, the prognosis is good. When hyperpyrexia or in fact any temperature above 102° F., is found, it must be energetically treated. The temperature must always be taken in cases of *status epilepticus*, because often there is no sign suggestive of pyrexia until the thermometer is used. Give no food. Get fluid into the patient. Refrain from poisoning him with repeated doses of chloral or amylene hydrate. These are Dr. McDougall's principles.

### A Living Foreign Body in the Throat.

Mr. Mukerji, sub-assistant surgeon of the Koondi Dispensary, reports in the *Indian Medical Journal* the case of a Mahomedan boy, aged seven, who, while fishing with his companions, caught a kai-fish, and was advised by one of the latter to keep it in his mouth. The fish slipped down his throat and became impacted there. It was about 4 inches long and one inch broad and had thorny fins. On admission to the dispensary the boy was suffering from urgent dyspnœa with cyanosis and blood-stained vomiting. After gagging the patient and various futile attempts to dislodge the fish by manipulations with the fingers and forceps, the author inserted a curved needle into the tail of the fish and with the help of this succeeded in removing the foreign body. Relief was instantaneous. There was some laceration of the soft parts of the throat which made swallowing difficult for two days. This was treated with an immediate application of silver nitrate solution of a strength of 20 grains to the ounce followed by eight-hourly applications of glycerine and perchloride of iron. The boy was discharged on the fourth day cured.

### Self-Drugging in the United States.

Dr. Lyman F. Kebler contributes an article on self-drugging and legislation to the *Monthly Cyclopædia and Medical Bulletin*. He states that in spite of restrictive legislation and the warnings of the foremost medical men, the amount of opium, morphine, and their derivatives *per caput* imported into, and consumed in, the United States has doubled during the past forty years. Other habit-forming drugs are largely used also. Thus 150,000 ounces of cocaine or its salts are consumed annually, an amount quite ten times as great as is actually needed for legitimate medical purposes. Some 500,000 pounds of opium are imported annually, whereas a generous allowance for medicinal use would dispose of less than 100,000 pounds. There are at least a million drug *habitués* in the United States, and some authorities state that as many as four millions of the whole population are thus addicted. The author has addressed a series of questions to physicians and sanitarians with a view to discovering the commonest drugs employed by those addicted to drug-taking, the cause of the victims becoming so addicted, whether the habit was decreasing or increasing, and what were the occupations of the victims. Among replies received as to the measures that should be adopted to overcome the evil were the following: (1) Legislation prohibiting the sale of such drugs except on a physician's order; (2) No drug to be supplied twice on the same order; (3) Education of the public, and especially of the young; (4) Government and State institutions for *habitués;* (5) Stimulation of physicians and druggists to prevent drug habits; (6) Make illegal sale a penal offence (7) Continue present laws as to labelling; (8) Banish from trade all patent medicines containing habit-forming drugs.

